# Short-Term Opioid Treatment of Acute Locomotor Pain in Older Adults: Comparison of Effectiveness and Safety between Tramadol and Oxycodone: A Randomized Trial

**DOI:** 10.3390/geriatrics9020046

**Published:** 2024-04-05

**Authors:** Wim Henri Janssens, Pauwelijn Verhoestraete, Ruth D. Piers, Nele J. Van Den Noortgate

**Affiliations:** Department of Geriatrics, University Hospital Ghent, C. Heymanslaan 10, 9000 Ghent, Belgium

**Keywords:** opioids, older adults, acute locomotor pain, tramadol, oxycodone

## Abstract

Introduction: We conducted a head-to-head comparison of step 2 (tramadol) and step 3 (oxycodone) of the WHO pain ladder in older adults with moderate to severe acute locomotor pain. Materials and methods: Multi-center prospective randomized study. Patients were 70 years or older, admitted to the acute geriatric ward of three hospitals, suffering from acute moderate to severe locomotor pain, and opioid-naive. Patients were randomized into two treatment groups: tramadol versus oxycodone. The Consort reporting guidelines were used. Results: Forty-nine patients were included. Mean numeric rating scale (NRS) decreased significantly between day 0 and 2 of the inclusion in both groups. A sustained significant decrease in mean NRS was seen at day 7 in both groups. Nausea was significantly more prevalent in the tramadol group, with a trend towards a higher prevalence of delirium and falls and three serious adverse events in the same group. Conclusions: Opioid therapy may be considered as a short-term effective treatment for moderate to severe acute locomotor pain in older adults. Oxycodone may possibly be preferred for safety reasons. These results can have implications for geriatric practice, showing that opioids for treatment of acute moderate to severe locomotor pain in older patients are effective and safe if carefully monitored for side effects. Opioid therapy may be considered as a short-term treatment for moderate to severe acute locomotor pain in older adults, if carefully monitored for (side) effects, while oxycodone may possibly be preferred for safety reasons. These results can have implications for daily practice in geriatric, orthopedic, and orthogeriatric wards, as well as in terminal care, more precisely for the treatment of moderate to severe acute locomotor pain in older adults.

## 1. Introduction

Pain is frequent in every age category, but localization, pattern, and consequences of pain change with increasing age. Because pain, acute as well as chronic pain, in older persons frequently has an orthopedic cause (e.g., fracture or osteo-arthritis), older patients report more frequently pain in joints and extremities compared to younger people [1,2]. The older the patient, the greater impact (locomotor) pain has on activities of daily living and care dependency [3]. Acute pain is present in more than two thirds of patients hospitalized on an acute geriatric ward (including orthogeriatric wards), but less than half of them are well treated [4]. In addition, opioids are also a key element in terminal care for treatment of acute as well as chronic pain [5,6,7]. Although lack of adequate treatment can be due to different assumptions such as pain being part of old age or to the influence of cognitive impairment on recognizing and expressing pain, the limited use of adequate pain relievers such as opioids is one of the main reasons for this.

Despite opioids being widely known as potent pain relievers and being put forward to manage pain in older adults in a stepwise approach [8,9], their use is often limited because of side effects (or fear of side effects), especially in older individuals [10,11,12]. A major challenge when treating older patients with opioids is indeed the risk of adverse effects, such as sedation, respiratory depression, decline in liver function, neurotoxicity with seizures, constipation, nausea and vomitus, risk of falling, and urinary retention [13,14]. The increased risk of side effects in older adults is mostly due to alterations in pharmacokinetics occurring with normal aging, polypharmacy, and multimorbidity [15]. Understanding appropriate indications and being able to recognize and manage side effects, as well as selection of the correct opioid based on patient characteristics, effects, and side effects, are key elements in treating older patients with acute and chronic pain [16,17,18]. However, there is a lack of evidence about short-term use of opioids in older adults, and especially about which opioid, weak or strong, has the best safety/effectiveness profile. This makes it difficult to decide which opioid is the best choice for treating acute moderate to severe pain in older adults.

Weak opioids, such as tramadol, are known to cause delirium, due to the existence of metabolites with anticholinergic effects. Therefore, use of tramadol in older adults is controversial [17]. However, tramadol is widely used as a weak opioid, not only in Belgium [19] but also in other countries, as confirmed by a meta-analysis by Furlan et al. [20]. On the other side, oxycodone, as a strong opioid, is one of the preferred evidence-based choices for opioid therapy in older adults [15]. However, literature about a head-to-head comparison of tramadol and oxycodone in older adults is scarce. Therefore, the purpose of this study is to compare effectiveness and safety of tramadol versus oxycodone in a short-term treatment schedule in older patients with acute moderate to severe locomotor pain.

## 2. Materials and Methods

### 2.1. Design and Study Population

We conducted a multi-center prospective randomized study on patients admitted to the acute geriatric ward of 3 Belgian hospitals (1 university hospital and 2 general hospitals) during a 4-year period.

Patients included by the treating geriatrician (working on the relevant department) were 70 years or older, admitted to the acute geriatric ward with acute (less than 72 h) moderate to severe locomotor pain (at least one report of locomotor pain on the day of inclusion of numeric rating scale (NRS) ≥ 5). Exclusion criteria were treatment with opioids during at least two consecutive weeks in six months prior to the inclusion, need for surgical intervention, other types of pain (inflammatory pain, malignant pain, pain due to ischemia, purely neuropathic pain, or chronic pain without flare-up), end-of-life, severe renal failure (chronic kidney disease IV-V), and liver function decline (level of transaminases higher than 2 times reference value).

Patients were randomized into 2 treatment groups using the SAS system. The first group was treated with tramadol extended release (ER) 50 mg twice a day, with tramadol instant release (IR) 50 mg as rescue medication in case of breakthrough pain, with a maximum of four times a day (step 2 of the WHO ladder, weak opioid). The second group was treated with oxycodone extended release (ER) 5 mg twice a day, with oxycodone instant release (IR) 5 mg as rescue medication, with a maximum of six times a day (step 3 of the WHO ladder, strong opioid). Patients were followed during seven days.

Written informed consent was obtained. Patients not being able to sign the written informed consent were excluded from inclusion.

### 2.2. Variables

At inclusion, the following data were collected: gender, age, living situation, Katz index of Dependency in Activities of daily living [21], geriatric risk profile (GRP) (i.e., version of Triage Risk Screening Tool (TRST) [22]), etiology and localization of the pain, and co-medication.

Rescue medication was noted. Three times a day, the pain level was obtained (NRS, 0 to 10), as well as the presence of nausea. Bowel movements, urinary retention, confusion, or falls were listed. Katz score was calculated on day 0 and day 7. An electrocardiogram was obtained on day 0 and day 1 to check for QT lengthening. The primary outcome was the decrease in mean NRS after 2 and 7 days. Secondary outcomes were time of achieving adequate pain control, use of rescue medication, time of last acute pain flare-up, and evolution of functionality.

### 2.3. Statistics

The calculated sample size was 60 patients in each group for the primary outcome. More specifically, starting from the hypothesis that a difference between weak and strong opioids exists, an equivalence test of means using the confidence interval approach (95%CI constructed) on data from a parallel-group design with sample sizes of 52 in each group achieves 90% power when the true difference between the means is 0, the standard deviation is 1.4, and the equivalence limits are −1 and 1. Taking a drop-out of 8 patients in each group into account (statistical/theoretical assumption), the calculated sample size was 60 patients in each group.

Statistical analysis was performed using the SPSS 27 program (Statistical Package for the Social Sciences—Windows). Differences between both treatment groups were calculated using the Pearson chi-square test for categorical variables and the Mann–Whitney U test for continuous variables. The paired Student’s *t*-test was used to evaluate the evolution of the NRS in both groups and the Pearson chi-square test to evaluate the differences in side effects and the evolution in Katz score between both groups. *p*-levels (α) were considered significant if ≤0.05.

The Consort reporting guidelines were used [23].

## 3. Results

Forty-nine patients were included (from October 2016 to September 2020), 25 in the tramadol treatment group and 24 in the oxycodone group. Table 1 provides an overview of patient characteristics.

### 3.1. Primary Outcome

Mean NRS decreased significantly between day 0 and 2 of the inclusion, both in the tramadol group (NRS 3.8 (day 0) vs. 2.1 (day 2), *p* < 0.001) and in the oxycodone group (NRS 3.8 (day 0) vs. 2.0 (day 2), *p* < 0.001).

A sustained significant decrease in mean NRS was seen at day 7, both in the tramadol group (NRS 3.8 (day 0) vs. 1.2 (day 7), *p* < 0.001) as well as in the oxycodone group (NRS 3.8 (day 0) vs. 1.6 (day 7), *p* < 0.001).

Results are shown in Table 2.

### 3.2. Secondary Outcomes

Concerning the time of achieving adequate pain control (mean NRS ≤ 1), no significant differences between the treatment groups were detected (day 4.2 (SD 2.8) versus day 3.7 (SD 3.0) in tramadol group and oxycodone group, respectively, *p* = 0.484).

An average of 4 rescue analgesics were given in the tramadol group (range 0–13), versus 3.5 in the oxycodone group (range 0–14). The average daily use of rescue medication (all patients included) was 0.49 pills (0.52 in the tramadol group, compared to 0.52 in the oxycodone group, *p* = 0.58).

The timing in days of the last acute pain flare-up, seen as a one-time NRS value of ≥5, did not differ significantly between both treatment groups (tramadol group mean of 3.3 days (SD 3.1) versus oxycodone group mean of 4.1 days (SD 3.2), *p* = 0.309).

Evolution of functionality between day 0 and day 7 was investigated, measured by the Katz scale (insufficient data in nine patients). Functionality was preserved in 12 of the 20 patients treated in the tramadol group (60%) and in 15 of the 20 patients in the oxycodone group (75%) (*p* = 0.058).

Results are shown in Table 3.

### 3.3. Side Effects

Prevalence of side effects is listed in Table 4.

A total of 11 out of 49 patients did not complete the study (7 in the tramadol group and 4 in the oxycodone group) (Table 5). In the tramadol group, one patient did not complete the study because of an acute pulmonary edema on day 2, one patient because of convulsions on day 2, and three patients because of delirium (on day 2, 3, and 6). In one patient in the tramadol group, treatment was discontinued on day 6 because of insufficient efficacy, with the need of changing the treatment schedule. A last patient in the tramadol group dropped out because of the combination of insufficient efficacy and delirium on day 5. In the oxycodone group, one patient did not complete the study because of delirium (day 3) and one patient because of swallowing difficulties, with the patient no longer being able to take the study medication. One patient in the oxycodone group dropped out because of an unspecified reason (day 3) and one patient because of early discharge, with no further need for treatment (day 7).

In 3 out of 49 patients, serious adverse events (SAE) were registered during the study period, all in the tramadol group. One patient, with a history of diastolic and multivalvular heart failure, developed an acute pulmonary edema at day 2 of the follow-up. The second patient developed convulsions of the upper limbs at day 2 of the follow-up. A CT scan of the brain excluded an acute ischemic event and bleeding. The treatment with tramadol was interrupted immediately and, 24 h later, the convulsions stopped. Acute delirium was also registered as an SAE in one patient.

## 4. Discussion

In this study, we compared the effectiveness and safety of tramadol and oxycodone in the treatment of acute moderate to severe locomotor pain in older adults admitted to the acute geriatric ward. Acute orthopedic problems are a frequent cause of pain and hospitalization of older patients on acute geriatric and orthogeriatric wards, even when there is no need for surgical intervention.

Both opioids had similar effects on pain in this cohort. More specifically, no difference was noted in time needed to reach analgesic effect, time needed to achieve complete pain control, or need for additional analgesics for breakthrough pain. This is in concordance with earlier study results, where, admittedly in a study population aged 36–88 years, no significant difference was found between weak (tramadol) and strong (buprenorphine) opioids [24]. In a meta-analysis by Furlan et al., weak and strong opioids outperformed placebo in chronic noncancer pain [20]. However, another systematic review on the use of opioids in treating musculoskeletal pain showed only a small effect on pain compared to placebo, with a higher risk for adverse events [25]. This was confirmed in other systematic reviews on the use of opioids in osteo-arthritis [26,27]. However, in the studies included in those systematic reviews, opioids were used for treatment of chronic pain (e.g., osteo-arthritis) and often in younger patients. The strongest effect on pain in one meta-analysis was seen in the first 2 to 4 weeks of treatment, with decreasing benefits the following weeks, suggesting higher benefits of opioids for acute pain treatment [27]. Only a few previous studies compared tramadol to oxycodone, with comparable results. However, patients in those studies were much younger and indications were different (only postoperative patients were included in those studies) [28,29]. Despite the fact that data on the use of tramadol in older individuals are scarce, tramadol is still widely used in geriatric patients, especially in those with an acute indication, often with a locomotor origin. In a study on patients admitted to the geriatric ward of 14 Belgian hospitals, 57.9% of patients treated with opioids received tramadol, 78.9% of them because of an acute event, mainly of locomotor origin (fracture as well as nonfracture) [19].

In our study, treatment duration was 7 days. It would be useful to evaluate the effect and the side effects of opioids for a longer period. However, taking into account the fact that significant pain release was obtained after 2 days of treatment and a sustained pain relief was achieved at day 7, one could argue that, in case of an acute event, treatment with opioids can be short-term and can often be reduced and even stopped after a few days. In this view, it is important to re-evaluate the effect and the need for opioid treatment regularly, in order to avoid more side effects.

Nausea was significantly more prevalent in patients treated with tramadol. In the relevant literature, a head-to-head comparison between both opioids in older adults is not available. Some studies found a lower incidence of nausea as a side effect of opioid use, without distinction between weak or strong opioids and not specifically in older adults [17,30]. Constipation, the most common side effect, was equally present in both treatment groups. Other studies showed a comparable [31] or a lower [24,32] incidence of constipation, whether in patients treated with tramadol or with transdermal buprenorphine. However, this could be due to another definition of constipation in those studies. Other side effects, such as delirium and fall accidents, were more frequent in the tramadol group but without statistical significance being reached (due to small numbers). While pain per se can be the cause of delirium, treatment reluctance out of fear for delirium as a side effect is not justifiable [33]. Furthermore, an increased risk of side effects should always outweigh the potential risk of inadequately treating moderate to severe pain [34].

All serious adverse events occurred in the tramadol group. Convulsion is a known side effect of tramadol, by lowering the threshold for convulsions, and this is the reason why high-dose tramadol is contraindicated in patients suffering from epilepsy [35]. The appearance of pulmonary edema is more difficult to explain. Cardiovascular dysregulation with palpitations and tachycardia is a known side effect, however, especially occurring after intravenous administration and physical stress, as well as hypertension and hypertensive pulmonary edema [36]. An accidental coincidence cannot be excluded.

### Limitations and Strengths

The lack of power of this study is the most important limitation. One of the reasons for not obtaining the postulated sample size is the extensive list of exclusion criteria. Especially, exclusion of postoperative patients and of patients not being able to sign the written informed consent led to the exclusion of most patients with hip fractures and/or frailty and of patients suffering from dementia. Including those patients could probably influence the results concerning effectiveness and safety. In order to achieve more robust results useful for daily clinical practice, the sample size should be larger, including patients with surgical indications, patients with frailty and multiple co-morbidities, as well as patients suffering from dementia. By doing so, guidelines on acute pain treatment in older adults could be adjusted, instead of extrapolating guidelines largely from clinical experience and from studies on younger individuals or patients suffering from chronic pain. Finally, the influence of the COVID-19 pandemic during at least a part of the inclusion period, leading to a decrease in hospitalization of geriatric patients with non-COVID-related problems such as acute nonsurgical orthopedic problems, cannot be neglected. Regardless of these reasons, the barriers to include older adults in clinical trials, such as frailty and co-morbidities, are often extensive [37,38].

The focus of this study was on patients with acute locomotor pain. This group of patients represents a large proportion of patients with acute pain hospitalized on a geriatric ward. Nevertheless, pain due to other acute events occurring in older individuals, such as visceral pain, surgical indications, or neuropathic pain, can lead to the need for treating those patients with opioids. Because of the specificity of our study group, results cannot simply be extrapolated to those other patient groups.

Functionality of patients was only evaluated briefly. A more comprehensive evaluation of the impact of opioid use on activities of daily living and on physical and mental well-being could be the focus of further research, especially in those patients that are treated with opioids for a longer period.

The strength of this study lies in its implication for daily practice in geriatric, orthopedic, and orthogeriatric wards, showing that the use of opioids for treatment of acute moderate to severe locomotor pain in older patients is effective and safe if carefully monitored for (side) effects. This is the first study in older adults with moderate to severe acute locomotor pain with a head-to-head comparison of tramadol (step 2 of the WHO ladder) and oxycodone (step 3 of the WHO ladder). The higher prevalence of adverse effects that can negatively affect quality of life and quality of care, such as nausea, delirium, and convulsions, in patients treated with tramadol can also be taken into account when using opioids for treatment of pain in other settings, such as terminal care.

## 5. Conclusions

This is the first study in older adults with moderate to severe acute locomotor pain with a head-to-head comparison of tramadol (step 2 of the WHO ladder) and oxycodone (step 3 of the WHO ladder). Tramadol (weak opioid, step 2) and oxycodone (strong opioid, step 3) had similar effects on pain in a short-term treatment schedule in older patients with acute moderate to severe locomotor pain. As for side effects, nausea was more frequent in patients treated with tramadol compared to oxycodone, while there was a trend towards a higher frequency of delirium and fall accidents in the patients treated with tramadol. Opioid therapy may be considered as a short-term treatment for moderate to severe acute locomotor pain in older adults if carefully monitored for (side) effects. Oxycodone may possibly be preferred for safety reasons.

These results can have implications for daily practice in geriatric, orthopedic, and orthogeriatric wards, as well as in terminal care, including a better understanding of effectiveness and safety of opioids in short-term treatment for acute moderate to severe locomotor pain in geriatric patients.

## Figures and Tables

**Table 1:** Patient characteristics.

		All Patients (n = 49)	Tramadol Group (n = 25)	Oxycodone Group (n = 24)	*p*-Value
Mean age (years)		86 (range 74–97)	86 (range 77–97)	86 range 74–97)	0.833
Mean Geriatric Risk Profile *		2.7 (SD 1.03)	2.9 (SD 1.15)	2.5 (SD 0.85)	0.165
Living circumstances	At home	42 (85.7%)	20 (80.0%)	21 (87.5%)	0.544
	Assisted living facility	6 (12.2%)	4 (20.0%)	2 (8.3%)	
	Residential	1 (2.1%)	0 (0%)	1 (4.2%)	
Katz index of Independency in Activities of daily living°	O or A	47 (95.9%)	24 (96.0%)	23 (95.8%)	0.997
	B	2 (4.1%)	1 (4.0%)	1 (4.2%)	
Number of medications on day of inclusion		7.4 (SD 3.36)	7.4 (SD 2.76)	7.4 (SD 3.94)	0.860
Mean NRS on day of inclusion		3.8 (SD 1.40)	3.8 (SD 1.48)	3.8 (SD 1.34)	0.711
Etiology of pain	Fracture	38 (77.5%)	18 (72.0%)	20 (83.3%)	0.581
	Acute flare-up of known degenerative osteo-arthritis	2 (4.1%)	1 (4.0%)	1 (4.2%)	
	Bruise or contusion	9 (18.4%)	6 (24.0%)	3 (12.5%)	
Localization of pain	Trunk	24 (49.0%)	14 (56.0%)	10 (41.7%)	0.485
	Lower limbs	18 (36.7%)	9 (36.0%)	9 (37.5%)	
	Upper limbs	5 (10.2%)	1 (4.0%)	4 (16.7%)	
	Multiple locations	2 (4.1%)	1 (4.0%)	1 (4.2%)	

* Geriatric Risk Profile: score at 6 (presence of cognitive impairment, 2; lives alone or no available informal caregiver, 1; difficulty walking/transferring or recent falls, 1; previous hospitalization within past 3 months, 1; five or more medications, 1). Katz index of Independency in Activities of daily living (Dutch): O = independent; A = physically dependent for washing and/or dressing; B = physically dependent for washing, dressing, transfers, and toilet visit; C = physically totally dependent.

**Table 2:** Primary outcome.

Variable	Tramadol	*p*-Value	Oxycodone	*p*-Value
NRS day 0	3.8 (SD 1.5)	-	3.8 (SD 1.3)	-
NRS day 2	2.1 (SD 1.9)	<0.001 (day 2 vs. day 0)	2.0 (SD 1.6)	<0.001 (day 2 vs. day 0)
NRS day 7	1.2 (SD 1.1)	<0.001 (day 7 vs. day 0)	1.6 (SD 1.6)	<0.001 (day 7 vs. day 0)

**Table 3:** Secondary outcomes.

Variable	Tramadol	Oxycodone	*p*-Value
Time of achieving mean NRS ≤ 1	Day 4.2 (SD 2.8)	Day 3.7 (SD 3.0)	0.484
Average daily use of rescue medication (number of pills)	0.52	0.52	0.58
Day of last acute pain flare-up (day)	3.3 (SD 3.1)	4.1 (SD 3.2)	0.309
Functionality preserved at day 7 (% of patients)	60%	75%	0.058

**Table 4:** Prevalence of side effects.

	All Patients (n = 49)	Tramadol Group (n = 25)	Oxycodone Group (n = 24)	*p*-Value
Side effect—all (at least 1)	40 (82%)	21 (84%)	19 (79%)	0.73
Side effect—all (more than 1)	21 (43%)	15 (60%)	6 (25%)	0.01
Nausea—all	15 (31%)	12 (46%)	3 (13%)	0.011
Nausea—treatment (a)	7 (14%)	5 (21%)	2 (8%)	0.220
Urinary retention	4 (8%)	3 (12%)	1 (4%)	0.295
Constipation (b)	32 (65%)	16 (64%)	16 (67%)	1
Delirium—all (c)	15 (31%)	9 (36%)	6 (25%)	0.404
Delirium—treatment (d)	6 (12%)	4 (16%)	2 (8%)	0.413
Fall	4 (8%)	3 (12%)	1 (4%)	0.295
Liver function abnormalities (e)	2 (4%)	2 (8%)	0 (0%)	0.157
QT-prolongation (f)	2 (4%)	1 (4%)	1 (4%)	0.976
Convulsions	1 (2%)	1 (4%)	0 (0%)	0.322

(a) need for treatment with anti-emetics. (b) defined as 3 days without passage of stool despite the use of laxatives. (c) registered using DOS-scale. (d) need for treatment with psychotropic drugs. (e) de novo abnormal liver function tests at day 7 (doubling of SGOT). (f) detected 24 h after onset of treatment with opioids.

**Table 5:** Drop-outs from study: reasons for early termination and timing.

Group	Total Number of Drop-Outs	Reasons for Drop-Outs	Number of Drop-Outs
Tramadol	7	acute pulmonary edema (day 2)	1
		convulsions (day 2)	1
		delirium (day 2, 3, and 6)	3
		insufficient efficacy (day 6)	1
		insufficient efficacy and delirium (day 5)	1
Oxycodone	4	delirium (day 3)	1
		swallowing difficulty (day 3)	1
		unspecified (day 3)	1
		discharge, no more need (day 7)	1

## Data Availability

Full trial protocol and all data are accessible on request to the corresponding author.
